# Supplementary material for: Regulation of cerebral blood flow boosts precise brain targeting of vinpocetine-derived ionizable-lipidoid nanoparticles
Source: Nat Commun. 2024 May 11;15:3987. doi: 10.1038/s41467-024-48461-4 (PMC11088666; doi:10.1038/s41467-024-48461-4)
Supplement: Supplementary file 1 — Supplementary Information [file 41467_2024_48461_MOESM1_ESM.pdf]

Supplementary Materials

Bian X et al., Regulation of cerebral blood flow boosts brain penetration of vinpocetine-derived ionizable-lipidoid nanoparticles and their accumulation in lesions

Contents:

Materials and Methods	pp 2-6
Supplementary Figures S1 to S21	pp 7-27
Supplementary Tables S1 to S9	pp 28-36
Supplementary references	pp 37-38

Materials and Methods

Materials

Vinpocetine and tail chains were purchased from Bidepharm Co., Ltd. (Shanghai, China). DOPC (S01007), DSPC (S01005), DOPE (S03005), DMG-PEG_{2000} (O02005), cholesterol (O01001) and Dlin-MC3-DMA (AVT0003) were provided by A.V.T. Pharmaceutical Ltd. (Shanghai, China) (purity >98%). RiboGreen RNA Assay kits (GMS20129) were purchased from GENMED Co., Ltd. (Shanghai, China). siPDE1 (sequence: CCAAGGAGATTGAAGAATT), siBACE1 (sequence: GAACCUAUGCGAUGCGAAUTT), and siVEGF (sequence: GGAGUACCCUGAUGAGAUCTT) were purchased from Shanghai Sangon Biotechnology Co., Ltd. Rh123 (S19123) was purchased from Yuanye Biotechnology Co., Ltd. (Shanghai, China). Recombinant rat calcium calmodulin-dependent 3,5 cyclic nucleotide phosphodiesterase 1C (CSB-EP720512RA) was purchased from Cusabio (Wuhan, China). PDE1 Antibody (sc-376474, 200 μg/mL) was purchased from Santa Cruz Biotechnology (USA). Anti-BACE1 antibody (ab183612, 1:1000) was purchased from Abcam (Cambridge, MA, USA). Phospho-GSK3β (Tyr216) polyclonal antibody (44-604G, 1:1000) and phospho-Tau (Ser396) polyclonal antibody (44-752G, 1:1000) were purchased from Invitrogen (Carlsbad, CA, USA). Rabbit anti-beta amyloid 1-16 antibody (bs-10558R, 1:100) was purchased from Bioss Biotechnology Co., Ltd. (Beijing, China). GAPDH Polyclonal Antibody (10494-1-AP, 1:10000), Beta Actin Polyclonal antibody (20536-1-AP, 1:5000), Fluorescein (FITC)–conjugated Affinipure Goat Anti-Rabbit IgG(H+L) (SA00003-2, 1:100) and horseradish peroxidas (HRP)-conjugated Affinipure Goat Anti-Rabbit IgG (H+L) (SA00001-2, 1:5000) were purchased from Proteintech Group, Inc. (Wuhan, China). D-Luciferin (LUCK) was purchased from Gold Biotechnology Co., Ltd. (St Louis, USA). Hoechst 33342 (C1022) was purchased from Beyotime Biotechnology Co., Ltd. (Jiangsu, China). LysoRed (KGMP006) was purchased from KeyGEN Biotechnology Co., Ltd. (Jiangsu, China). eGFP mRNA (05297410) was purchased from Novoprotein Technology Co., Ltd. (Suzhou, China). SteadyPure Universal RNA Extraction Kit II, Evo M-MLV RT Mix Kit with gDNA Clean for qPCR Ver.2, and SYBR Green Premix Pro Taq HS qPCR Kit were purchased from Accurate Biotechnology Co., Ltd. (Hunan, China). All other reagents were analytical grade. ELISA kits were purchased from Enzyme-linked Biotechnology Co., Ltd. (Shanghai, China). Biochemical criterion kits were purchased from Grace Biotechnology Co., Ltd. (Suzhou, China).

Cell lines

bEnd.3 cells, A172 cells, and GL261-Luc cells were purchased from KeyGen Biotech (Jiangsu, China) and cultured in DMEM (KeyGen). PDE1$^{-/-}$ A172 cells were transfected with siPDE1 by using Lipofectamine 2000 (Invitrogen) according to the recommended protocol. One pair of siRNA targeting PDE1[1] was synthesized, and their knockdown efficiency was determined by flow cytometry. All of the above cells were cultured in complete medium containing FBS (Gibco). Cells were kept in an incubation chamber at 37°C and 5% CO_2 with a humidified atmosphere.

***C. neoformans* strains**

To generate luciferase-expressing strains of *C. neoformans* (wild-type H99), the coding domain of the luciferase gene LUC1 was amplified from the plasmid pCDW104-Luciferase with primers TL1943/1944 and cloned into the pTBL6 vector to generate the expression vector pTBL402 (PACTIN-LUC1). The resulting vector, linearized with XbaI, was precipitated onto 10-μL gold microcarrier beads (0.6 μm, Bio-Rad) and biolistically transformed into strain H99 ura5 as described previously[2]. Stable transformants were selected on SD-URA medium.

Mice

Female Balb/c mice (5-6 weeks, 18–22 g), male C57BL/6 mice (8 months, 28–32 g), and Sprague Dawley rats (180–220 g) were obtained from Chongqing Academy of Chinese Materia Medica (Chongqing, China), and male APP-PS-1 mice (2, 4, 6, 8 months) were obtained from Viewsolid Biotechnology Co., Ltd. (Beijing, China). In the mouse model of AD, APP/PS1 and WT-like mice injected with 200 μL of PBS were used as controls; the other treatment groups were given 1 mg of siRNA equiv/kg via tail vein injection every 2 days for 2 weeks. Therapy schedules for injection of LNP@siRNA and behavioral testing are shown in Figure 4a. All animals were raised in a pathogen-free laboratory animal environment, housed on a 12 h light/dark cycle at 22–24°C and 30–50% relative humidity. The laboratory animal facility has been accredited by the IACUC (Institutional Animal Care and Use Committee) of Southwest University Laboratory Animal Center (IACUC Issue No. IACUC-20221114-14). All animal experiments were conducted under the guidelines of the Ethical Review Committee of Experimental Animals at the Southwest

University of China.

Synthesis of VIP

A mixture of carboxylated head group (0.15 mmol), alkylated tails (3.18 mmol), ethyl (dimethylaminopropyl)-carbodiimide (EDC)·HCl (0.366 mmol) and DMAP (0.0162 mmol) was dissolved in 10 mL of dichloromethane and stirred at room temperature for 24 hours. The organic phase was then diluted with dichloromethane (5 mL) and washed four times with 2 M HCl (4 × 5 mL) and saline before being dried over sodium sulfate and concentrated to dryness. The resulting yellow oil was purified by using flash chromatography [acetone/hexane = 1/1 (v/v), dichloromethane/methyl alcohol = 1:3 (v/v)], and the final product was obtained through rotary evaporation. The lipidoid structures were confirmed by using ^{1}H nuclear magnetic resonance (NMR, Bruker, USA), and ^{13}C-NMR.

A5-B1-C4.2 Chemical Formula: $C_{97}H_{166}N_4O_4$. ^{1}H NMR (400 MHz, DMSO) δ 10.92 (s, 1H), 7.51 (dd, J = 29.9, 8.1 Hz, 2H), 7.19 (dd, J = 8.2, 6.9 Hz, 1H), 7.04 (t, J = 7.4 Hz, 1H), 5.41 - 5.16 (m, 16H), 3.68 (dd, J = 9.1, 7.2 Hz, 2H), 2.76 - 2.61 (m, 10H), 2.22 - 1.88 (m, 28H), 1.81 (q, J = 7.4 Hz, 3H), 1.53 - 1.15 (m, 79H), 1.07 (dt, J = 10.6, 7.0 Hz, 6H), 0.86 (t, J = 6.7 Hz, 13H), 0.64 (q, J = 8.4, 7.9 Hz, 4H). ^{13}C NMR (151 MHz, DMSO) δ 176.55, 139.52, 132.79, 130.83, 129.89, 127.17, 126.72, 122.40, 122.30, 115.71, 54.32, 50.73, 49.44, 38.17, 35.35, 34.03, 32.10, 31.83, 31.44, 31.37, 31.21, 30.61, 29.73, 28.32, 25.09, 21.74, 17.00, 11.06.

Molecular docking

Molecular docking was performed by using AutoDock Vina 1.1.2[3] to predict the binding patterns of small molecules and proteins. The PDE1 protein crystal structure was obtained from the Alphafold database (https://alphafold.ebi.ac.uk/), and the 3D structure of small molecule compounds was constructed with Chem3D 14.0, with the energy of the small molecules being minimized in the MMFF94 force field. Protein preparation was carried out by using PyMol 2.5.4[4] and involved removing hydrogenation, water molecules, and nonligand small molecules. A butt box was then defined to enclose the protein-active pocket, and ADFRsuite 1.0[5] was used to convert small molecules and receptor proteins in the PDBQT format. Docking was then performed with the conformation search detail set to 32 and all other parameters left at their default values.

The conformation with the top affinity score was selected as the correct conformation and visually analysed with PyMol 2.5.4.

Isolation and culture of primary brain microvessel endothelial cells

Primary BMECs were isolated as follows[6]. For brain tissue dissection, fresh brain tissue was obtained from SD rats (4-6 weeks old) and the brain was dissected to remove the meninges and choroid plexus. Brain tissue was then cut into small pieces and transferred to a 50-mL conical tube. For enzymatic digestion, a collagenase/dispase solution was added to the brain tissue pieces in the conical tube, which was incubated at 37°C for 30 minutes with shaking. After the incubation, the tissue pieces were triturated with a pipette to break them down into smaller fragments, after which a single-cell suspension was obtained by filtering the mixture through the cell strainer. For cell isolation, the cell suspension was centrifuged at 300 × *g* for 5 min to pellet the cells, and the pellets were resuspended in DMEM/F12 medium containing 10% FBS and penicillin/streptomycin. Cells were plated on a collagen-coated flask and incubated at 37°C with 5% CO_2. For cell culture, the medium was changed every 2-3 days and the cells were observed under a microscope to monitor their growth and morphology. After 7-10 days, the primary BMECs formed a monolayer of tightly packed cells.

Pharmacokinetics studies

LNP@DiD was injected into SD rats at a dose of 200 μg/kg by tail vein. Blood samples were collected from the orbit at different time points (0.25 h, 0.5 h, 1 h, 2 h, 4 h, 8 h, 12 h and 24 h). Approximately 200 μL of blood was collected from each rat and centrifuged at 4°C for 10 minutes (1000 × *g*). Then, 50 μL serum and 150 μL methanol were mixed by ultrasonication for 10 minutes to precipitate the protein and centrifuged at 4°C for 10 minutes (11000 × *g*) again. Finally, 50 μL of supernatant was analyzed with a 384-well black plate (Greiner, Germany) in a Multimode Microplate Reader (Tecan Infinite F200 pro, Switzerland) with excitation wavelengths of 595 nm and emission wavelengths of 665 nm. The pharmacokinetic parameters were calculated by PKsolver 2.0.10 software[7].

A5-B1-C4.2 and VIP was injected into SD rats at a dose of 5 mg/kg by tail vein. Blood samples were collected from the orbit at different time points (0.25 h, 0.5 h, 1 h, 2 h, 4 h, 6 h, 8 h,

12 h, 24 h, and 48 h). Approximately 200 μL of blood was collected from each rat and centrifuged at 4°C for 10 minutes (1000 × *g*). Then, 90 μL serum, 10 μL interior label (4-Nitro-1-naphthylamine, 10 μg/mL) and 400 μL methanol were mixed by ultrasonication for 10 minutes to precipitate the protein and centrifuged at 4°C for 10 minutes (11000 × *g*) again. Finally, 50 μL of supernatant was analyzed by HPLC. The pharmacokinetic parameters were calculated by PKsolver 2.0.10 software[7].

Regulation of cerebral blood flow

Cerebral blood flow was measured in mice by laser speckle flow imaging as described in the Methods. Mice were injected via tail vein with 5-hydroxytryptamine[8], dexmedetomidine[9], N^G-monomethyl-L-arginine[10], or noradrenaline[11]; doses were determined by monitoring cerebral blood flow to achieve similar reduction effect (40±5%) (Supplementary Table S6). After injection of these agents, mice were injected with VIP@DiD and fluorescence images were acquired 1 h, 2 h, and 6 h later by using a VISQUE in vivo Smart-LF System.

Evans blue dye exclusion tests

The fungal brain infection model was established as described above, and Evans blue (EB) dye extravasation was used to determine blood-brain barrier integrity[12, 13]. On days 1, 3, 5, and 7, Evans blue dye (dissolved in saline to a concentration of 2%) was given via tail vein at a dose of 2 mL/kg. After 2 hours, mice were deeply anesthetized and the chest was cut open, then 20 mL of saline and 20 ml of 4% paraformaldehyde were injected into the apex of the mouse heart through a catheter. The brain tissue was carefully isolated and weighed, transferred to a centrifuge tube containing 1 ml of PBS, and fully homogenized. After centrifugation at 4°C at a rotational speed of 10000 × *g* for 10 min, tissue homogenate supernatant (300 μL) was added to 700 μL of acetone, incubated at room temperature for 24 hours, and centrifuged at 4°C, at a rotational speed of 10000 × *g*, for 10 min. The absorbance value was measured with a Multimode Microplate Reader (BioTek Synergy H1, USA, Switzerland) at 620 nm.

Supplementary Figures

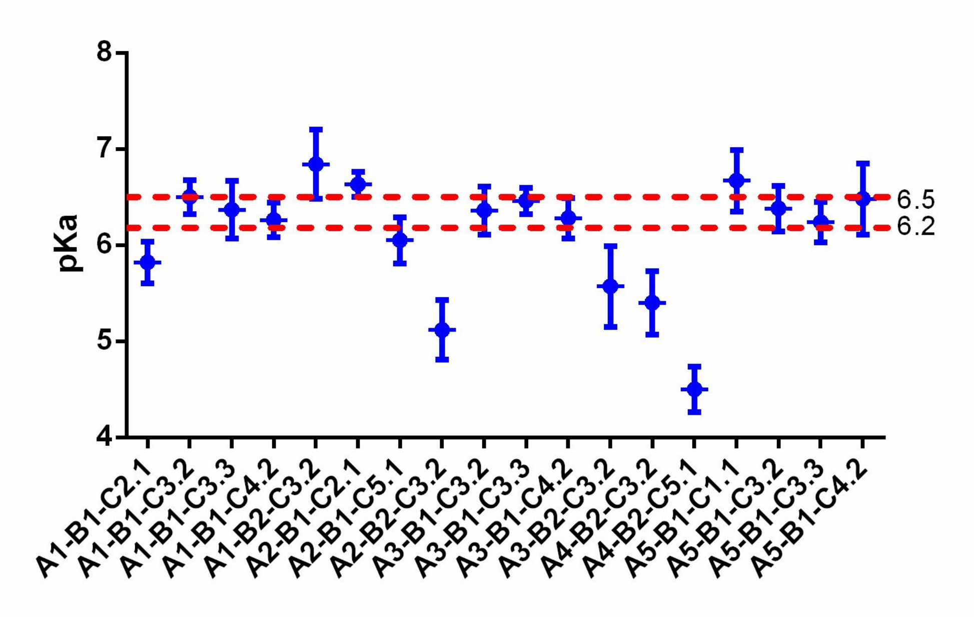

Supplementary Figure S1. Characterization of acid dissociation constant (pKa) of vinpocetine-derived ionizable-lipidoid nanoparticles (VIP) of <100 nm in diameter. Data are presented as means ± SD (n=3 biologically independent samples). Source data are provided as a Source Data file.

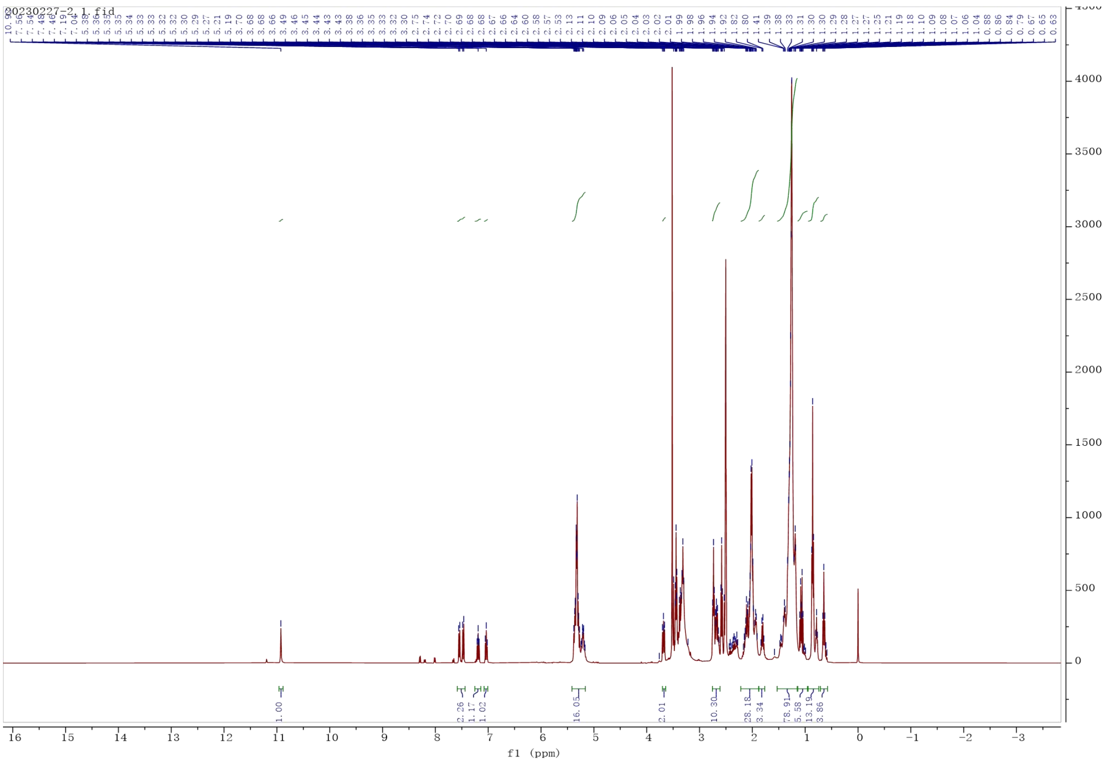

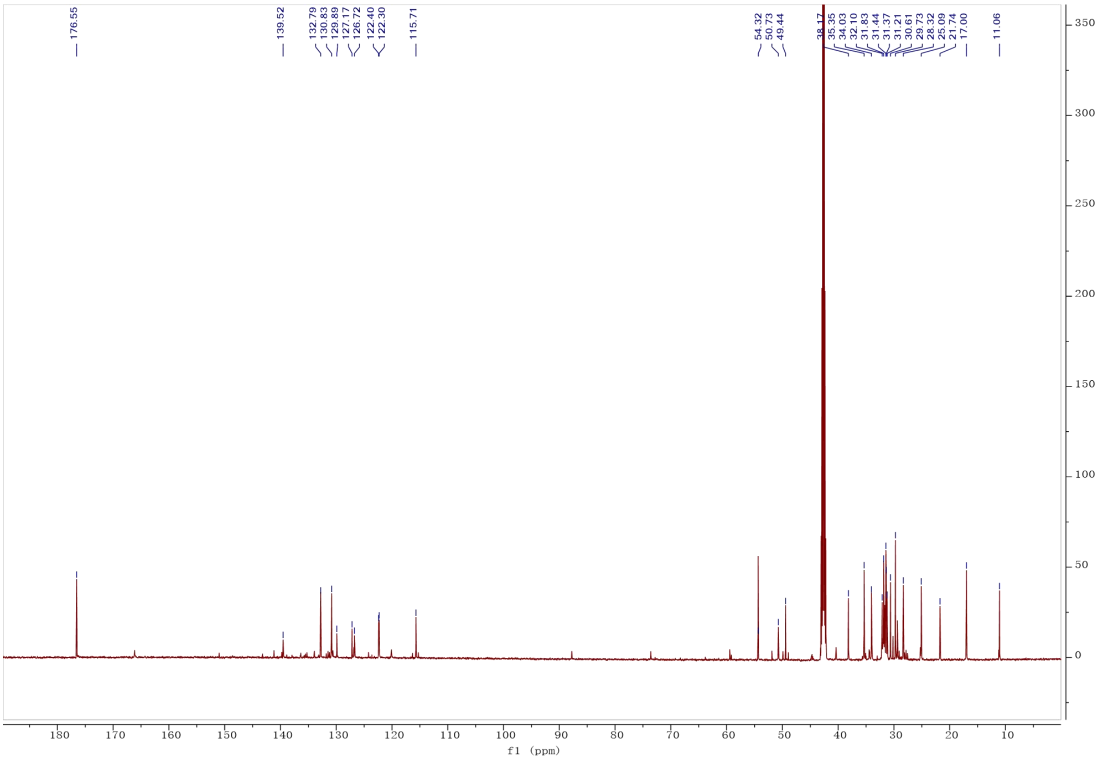

Supplementary Figure S2. ^{1}H NMR (D6-DMSO, 400 MHz) and ^{13}C NMR (D6-DMSO, 151 MHz) spectra of the representative ionizable lipid (A5-B1-C4.2).

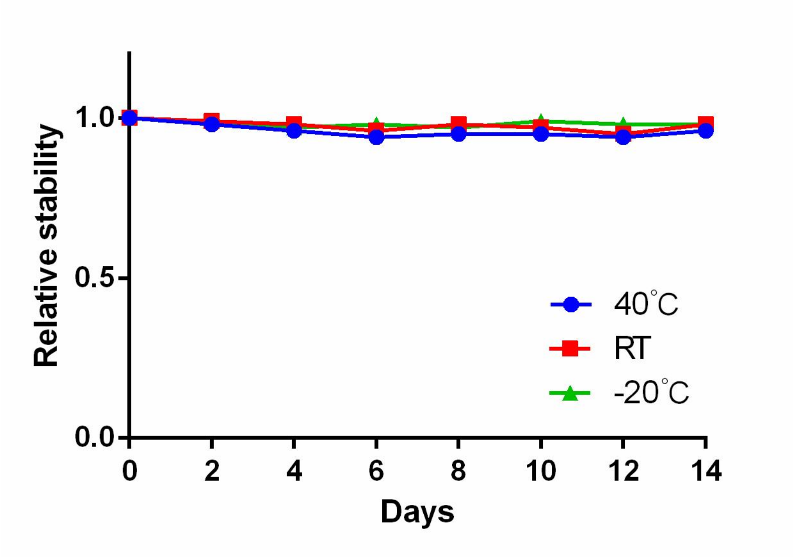

Supplementary Figure S3. Chemical stability of A5-B1-C4.2 over time. RT, room temperature. Source data are provided as a Source Data file.

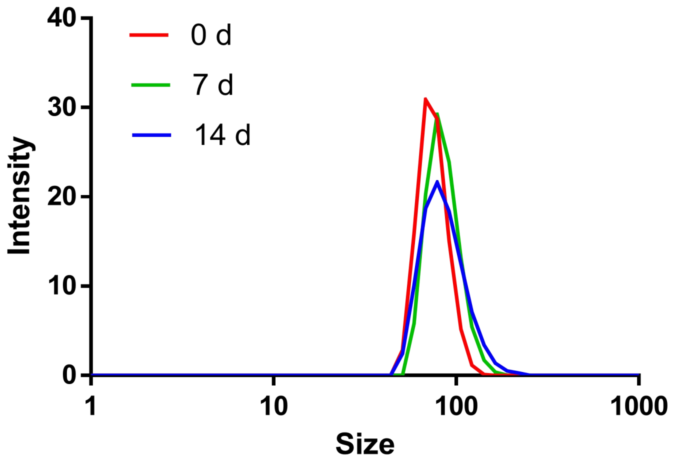

Supplementary Figure S4. Formulation stability of VIP@siRNA. Source data are provided as a Source Data file.

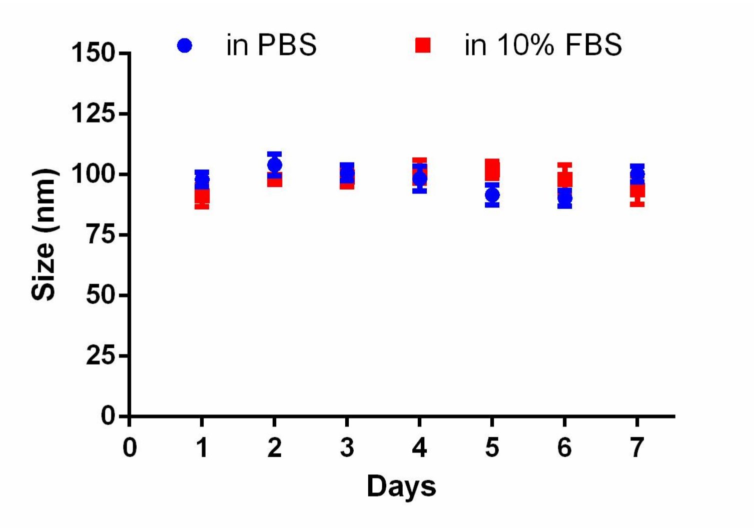

Supplementary Figure S5. Stability of VIP@siRNA in phosphate-buffered saline (PBS) and 10% fetal bovine serum (FBS) incubated at 37°C. Data are presented as means ± SD (n=3 biologically independent samples). Source data are provided as a Source Data file.

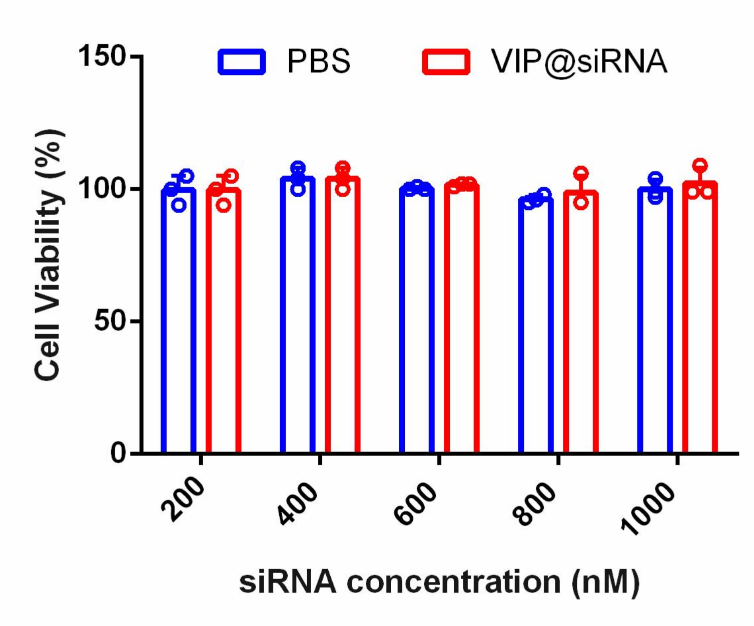

Supplementary Figure S6. *In vitro* MTT assay for viability of bEnd.3 cells treated with different concentrations of VIP@siRNA. Data are presented as means ± SD (n=3 biologically independent samples). Source data are provided as a Source Data file.

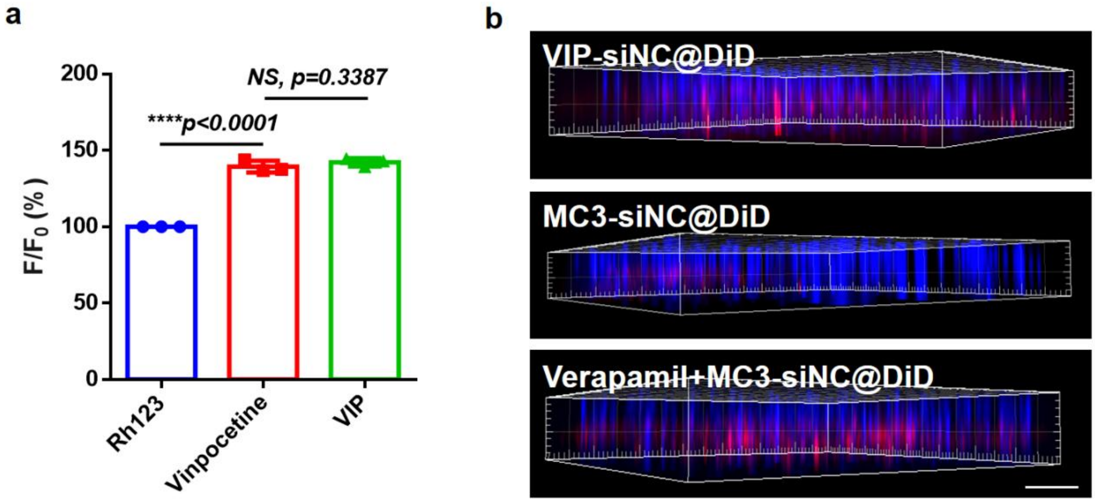

Supplementary Figure S7. Reversibility of P-gp inhibitor-induced effects on brain microvessel endothelial cell (BMEC) permeability. (**a**) BMECs were treated with a P-gp reversal agent (25 μmol/L) for 30 min, after which the agent was removed and cellular Rh123 accumulation was tested 90 min later. Data are presented as means ± SD (n=3 biologically independent samples). ****P<0.0001 , *NS* means no significance. Statistical significance was calculated with two-tailed unpaired *t* tests. Source data are provided as a Source Data file. (**b**) *In vitro* blood-brain barrier (BBB) model was established with a transwell assay to verify the permeability of LNP through the barrier, scale bar = 1000 μm. Images was collected after 4 h of incubation with VIP-siNC@DiD, MC3-siNC@DiD, and MC3-siNC@DiD pretreated with verapamil (50 μM), a known P-gp inhibitor, for 1 h. LNP@DiD were red. Nuclei were stained with DAPI (blue).

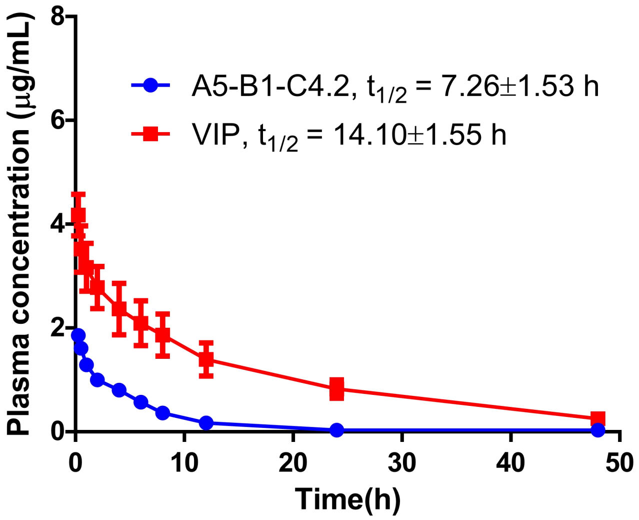

Supplementary Figure S8. Plasma concentrations of A5-B1-C4.2 in SD rats after intravenous administration of A5-B1-C4.2 or VIP. Data are presented as means ± SD (n=3 biologically independent samples). Source data are provided as a Source Data file.

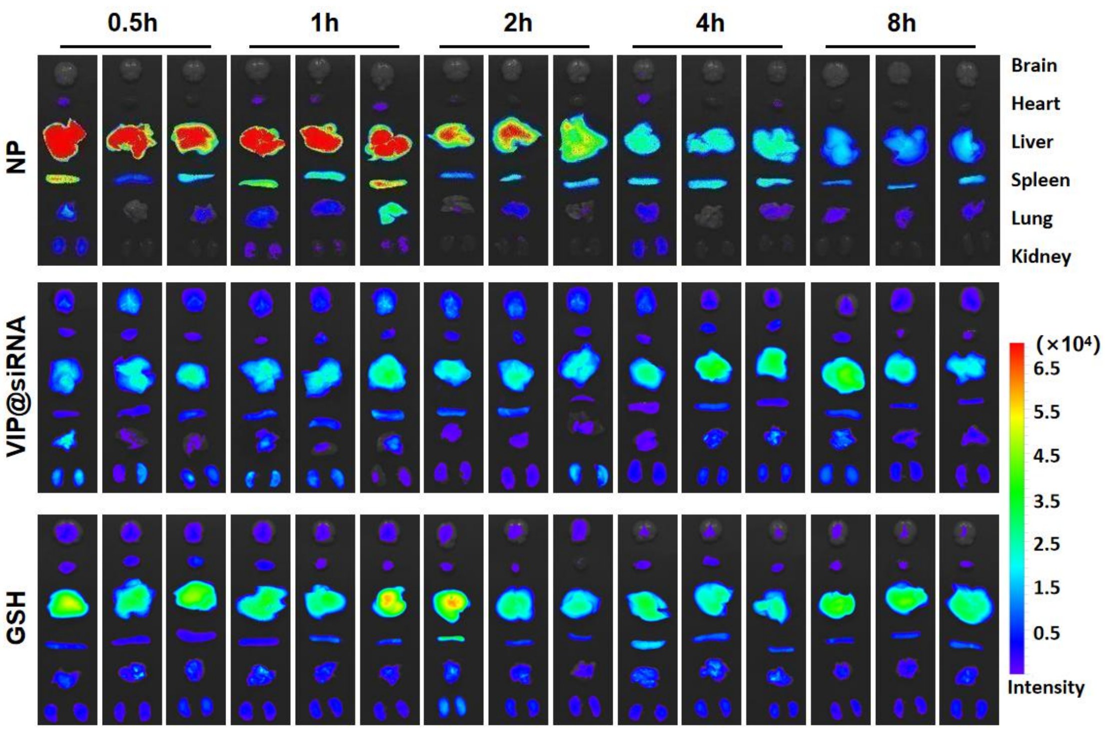

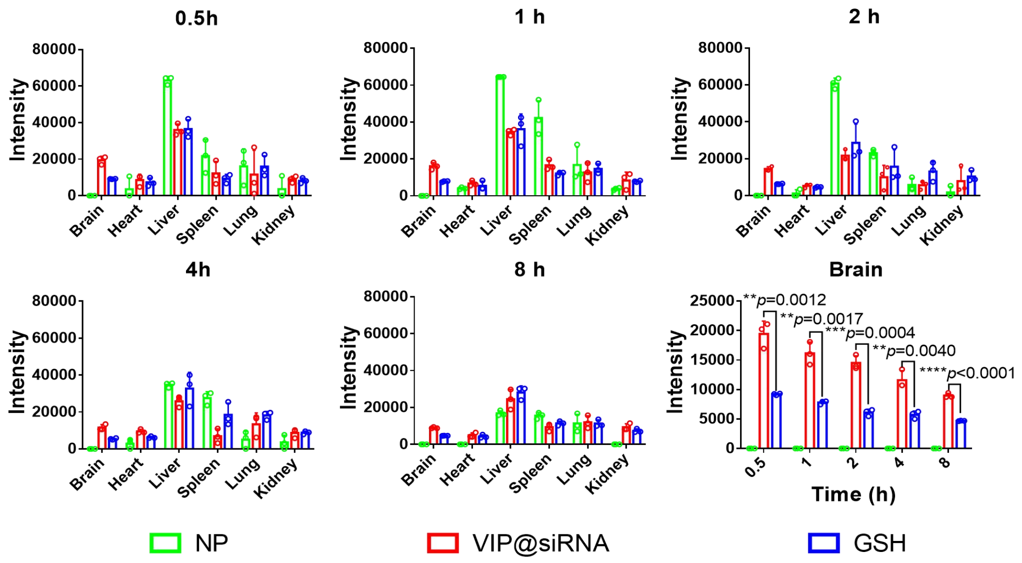

Supplementary Figure S9. *Ex vivo* fluorescence images of LNP@DiD at 0.5 h, 1 h, 2 h, 4 h, and 8 h after their injection into mice, measured by semi-quantitative analysis of fluorescence intensity. Data are presented as means ± SD (n=3 biologically independent samples). ***P*<0.01, ****P*<0.001, *****P*<0.0001. Statistical significance was calculated with two-tailed unpaired *t* tests. Source data are provided as a Source Data file.

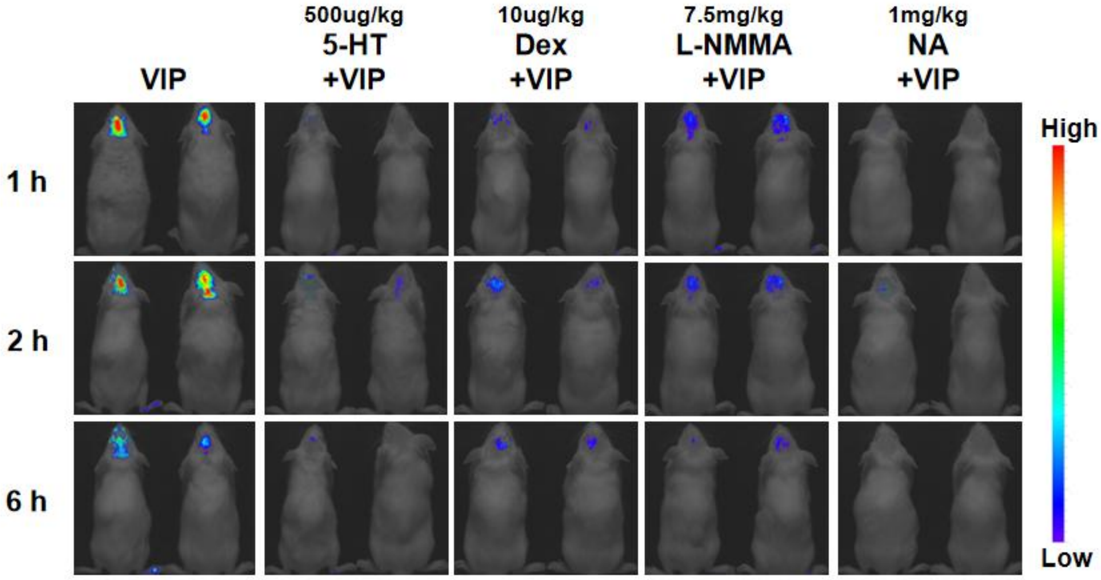

Supplementary Figure S10. *In vivo* fluorescence imaging after injection of VIP, alone or after pretreatment with the indicated agents [5-hydroxytryptamine (5-HT), dexmedetomidine (Dex), N^G-monomethyl-L-arginine (L-NMMA), and noradrenaline (NA)] at the indicated doses, followed by injection of VIP@DiD via tail vein. Fluorescence images were acquired at 1 h, 2 h, and 6 h after VIP@DiD injection.

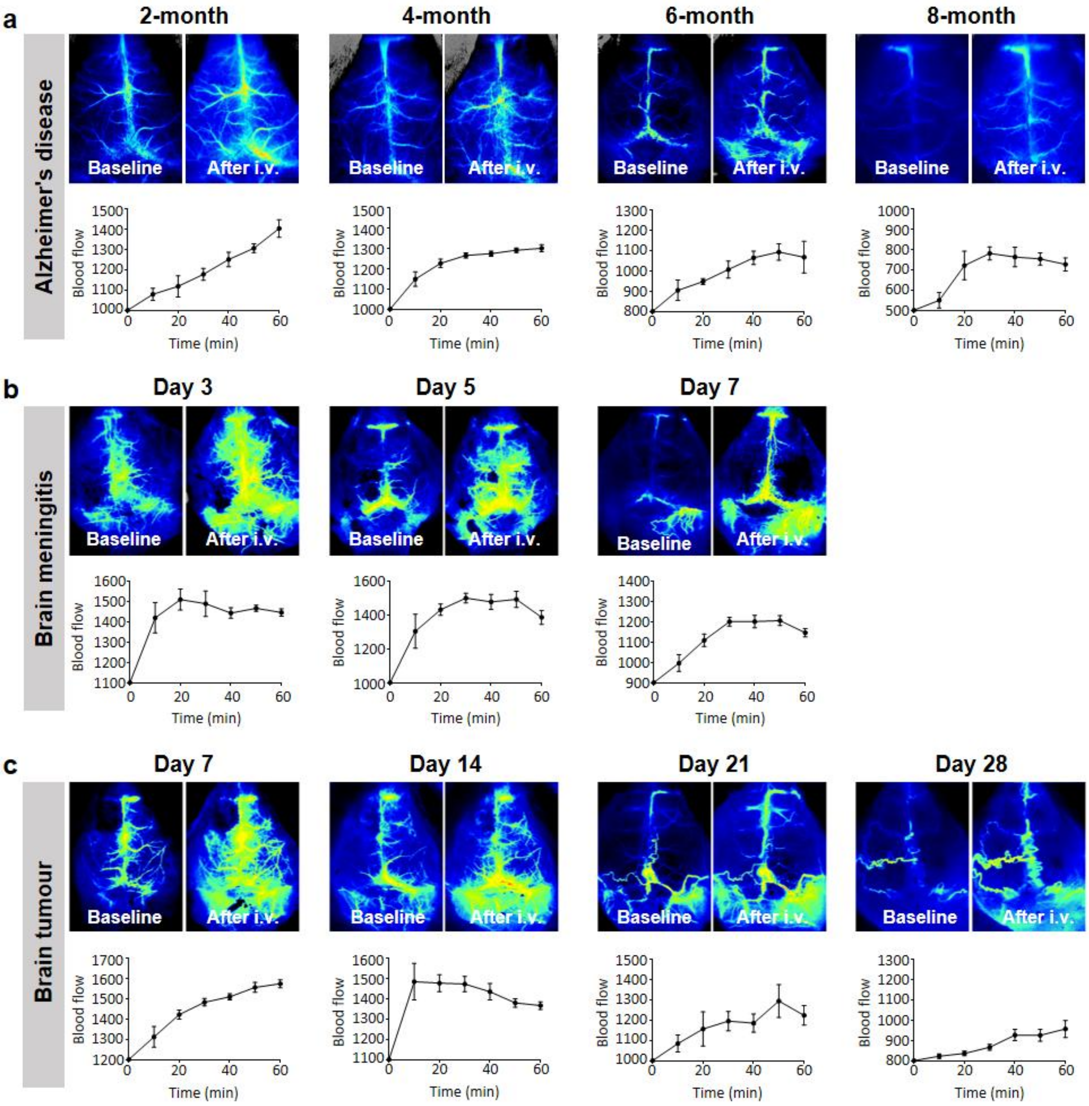

Supplementary Figure S11. Blood flow enhancement effect of VIP detected by using laser speckle flowgraphy in models of (**a**) Alzheimer disease, (**b**) brain meningitis, and (**c**) brain tumor. Data are presented as means ± SD, were obtained by measuring the blood perfusion unit every 10 minutes. Data are representative of two independent experiments with similar results. Source data are provided as a Source Data file.

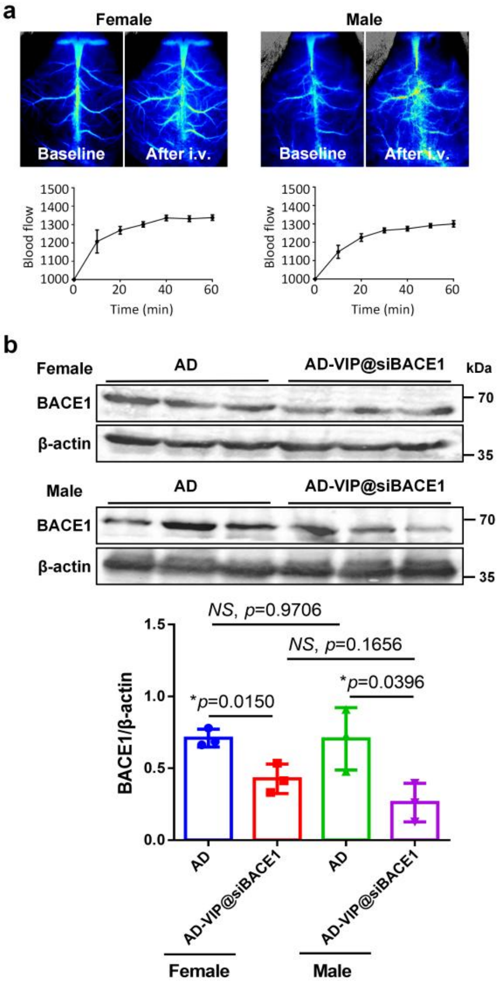

Supplementary Figure S12. Gender bias of cerebral blood flow enhancement and BACE1 protein silencing in the APP/PS1 mice model treated by the VIP system. (**a**) Blood flow enhancement effect of VIP on brain microvessels detected by using laser speckle flowgraphy in the AD models. Data are presented as means ± SD, were obtained by measuring the blood perfusion unit every 10 minutes. Data are representative of two independent experiments with similar results. (**b**) Western blot data for BACE1 protein expression in cortex from control APP/PS1 groups, and VIP@siBACE1-treated APP/PS1 mice. Quantification of western blotting analysis of BACE1 protein expression is shown relative β-actin. Data are presented as means ± SD (n=3 biologically independent samples). **P*<0.05, *NS* means no significance. Statistical significance was calculated with two-tailed unpaired *t* tests. Source data are provided as a Source Data file.

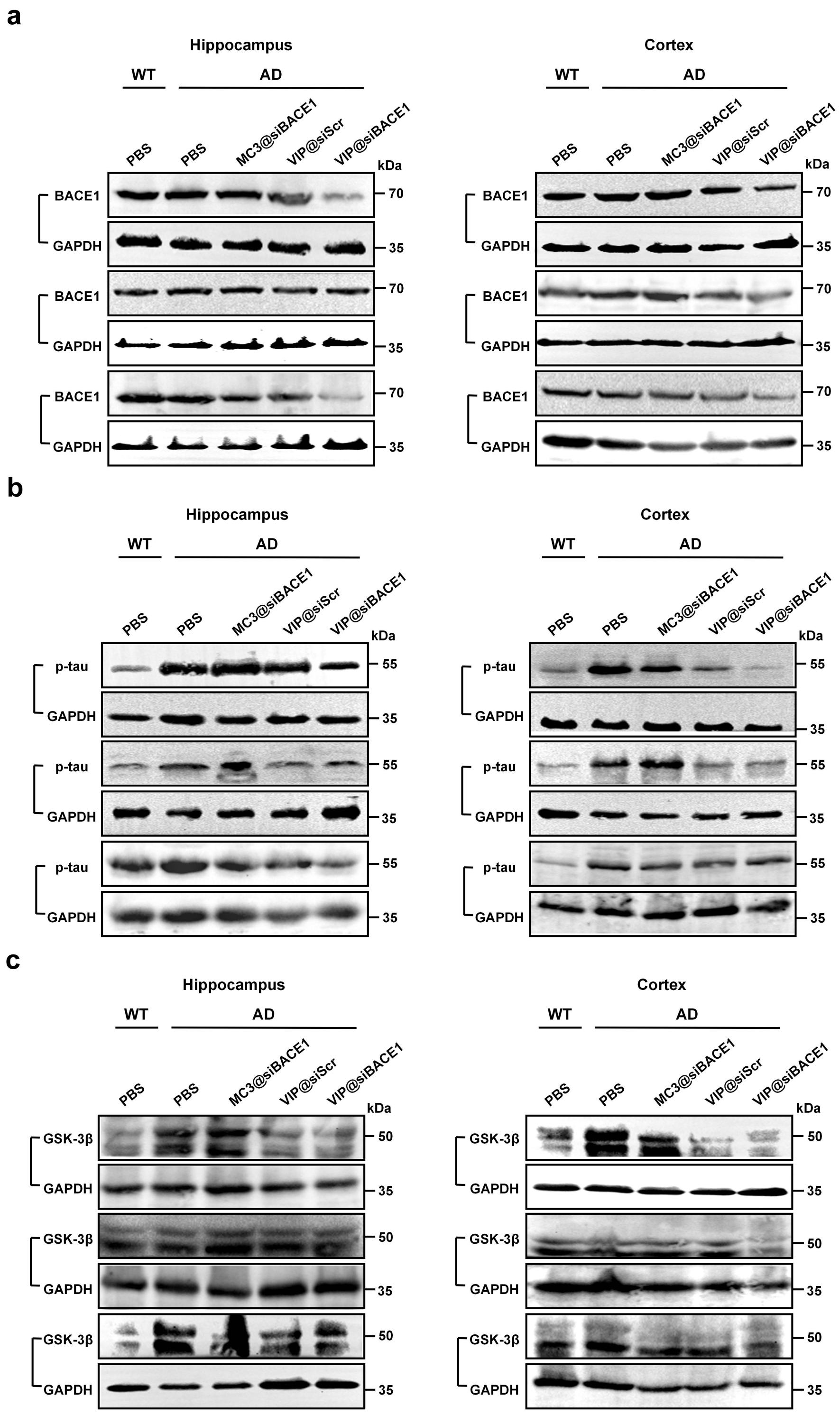

Supplementary Figure S13. Western blot data for expression of (**a**) BACE1, (**b**) p-tau, and (**c**) p-GSK3β protein in the hippocampus and cortex of LNP@siBACE1-treated APP/PS1 mice, control APP/PS1 mice, and WT mice. Quantification of the western blotting results for BACE1, p-tau, and p-GSK3β protein expression was relative to GAPDH. Source data are provided as a Source Data file.

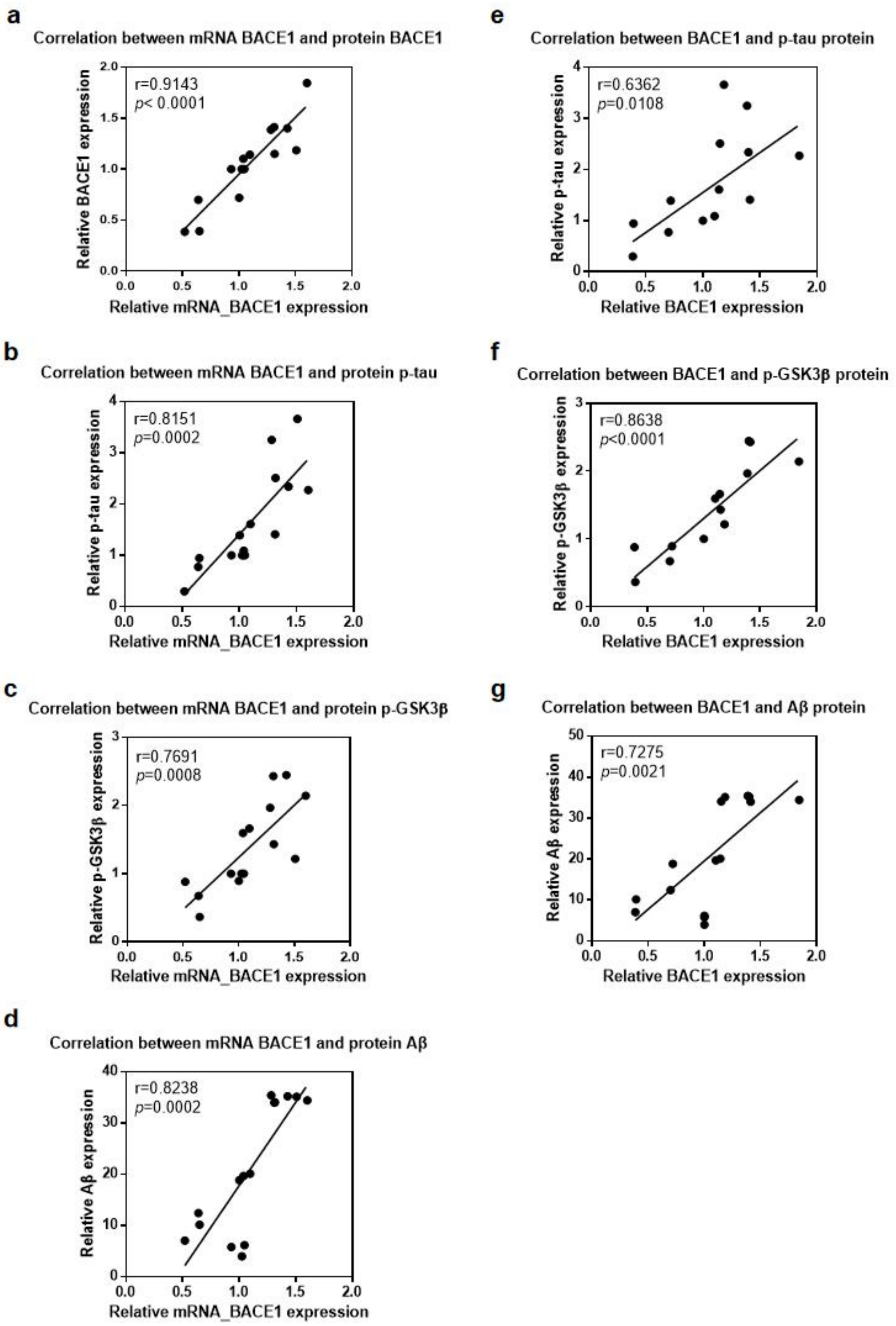

Supplementary Figure S14. (**a-d**) Correlations between BACE1 mRNA expression level and BACE1, p-tau, p-GSK3β, and Aβ protein expression levels. (**e-g**) Correlations between BACE1 protein expression level and p-tau, p-GSK3β, and Aβ protein expression levels. Association between BACE1 and p-tau, p-GSK3β, and Aβ protein expression in brain tissues was calculated using Pearson correlation test. Source data are provided as a Source Data file.

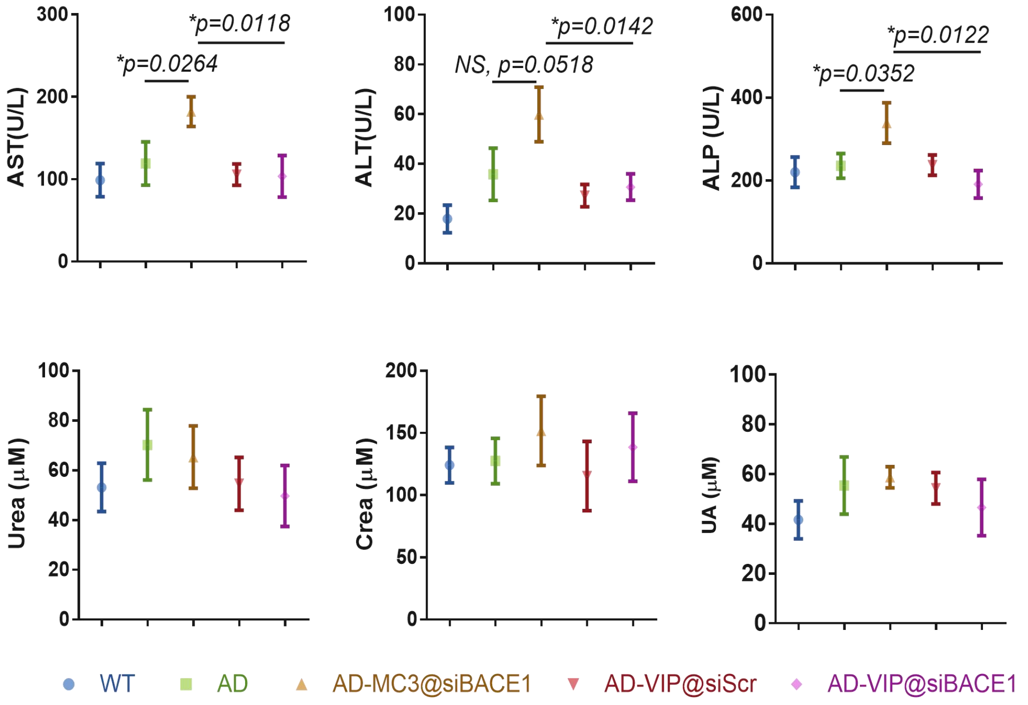

Supplementary Figure S15. Blood chemistry examinations. Assessment of plasma alanine aminotransferase (ALT), aspartate aminotransferase (AST), alkaline phosphatase (ALP), plasma urea (Urea), creatinine (Crea), and uric acid (UA) levels over the course of the 7 injections during the 2-week experiments. Data are presented as means ± SD (n=3 biologically independent samples). $*P<0.05$, *NS* means no significance. Statistical significance was calculated with two-tailed unpaired *t* tests. Source data are provided as a Source Data file.

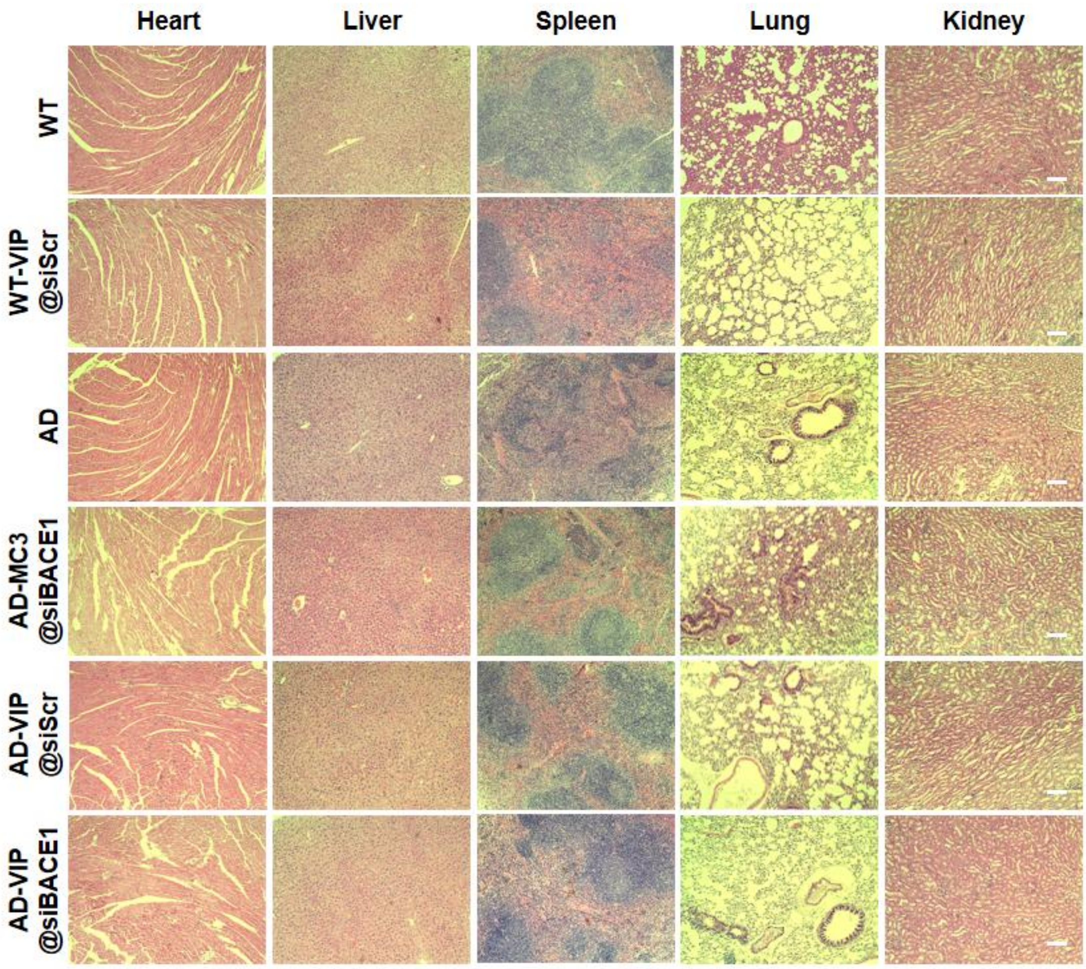

Supplementary Figure S16. Representative hematoxylin-and-eosin staining of sections from major organs from APP/PS1 and control WT mice treated with LNP@siBACE1 or PBS after the 2-week (7 injections) experiments. Scale bar = 100 µm. Data are representative of two independent experiments with similar results.

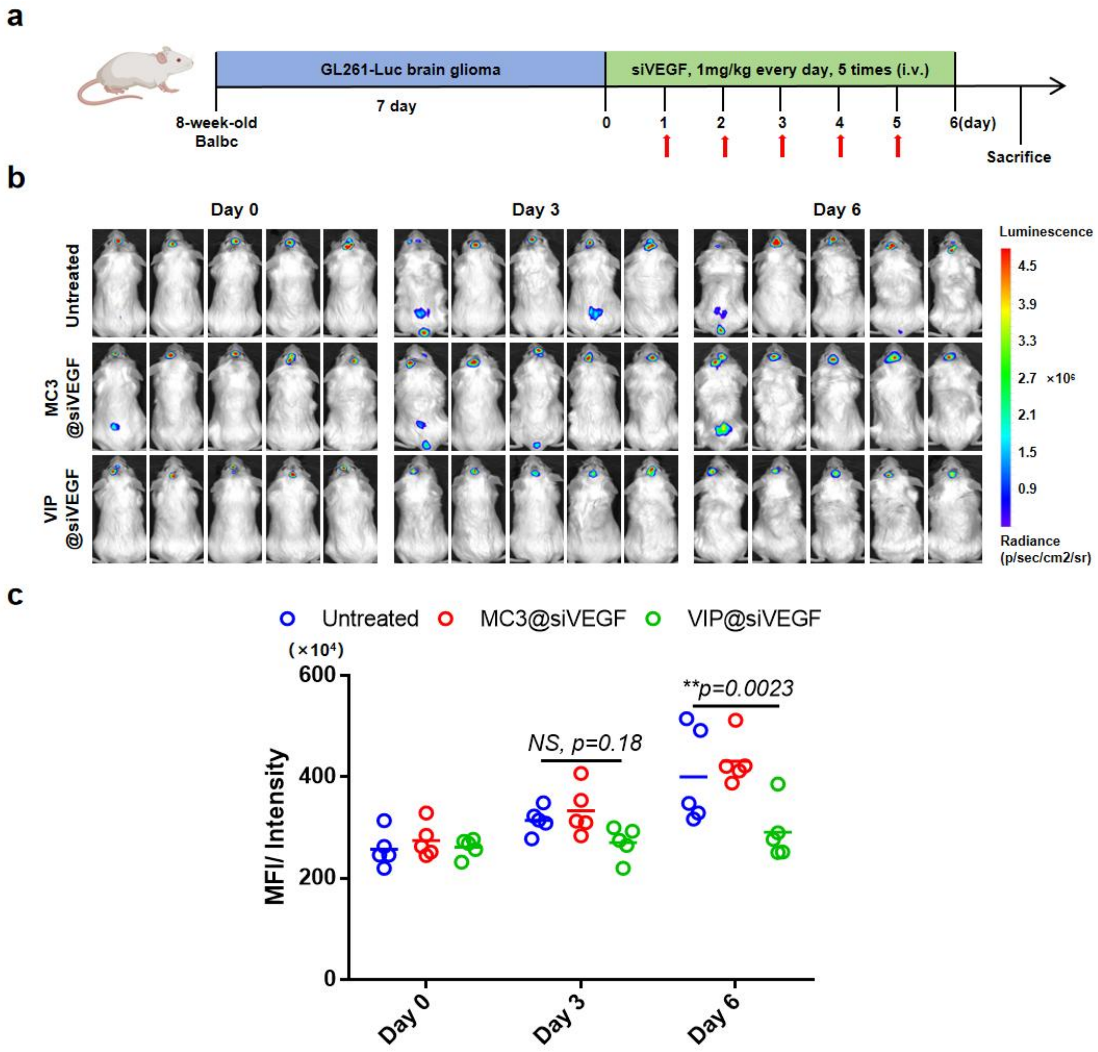

Supplementary Figure S17. (**a**) Schematic diagram of the experimental timeline. Mice were treated with LNP@siVEGF or PBS via tail vein injection every day (5 cycles). (Created with BioRender.com). (**b-c**) Fluorescence images and quantitative analysis of live imaging. Data are presented as means ± SD (n=5 biologically independent samples). $^{**}P$<0.01, *NS* means no significance. Statistical significance was calculated with multiple *t* tests. Source data are provided as a Source Data file.

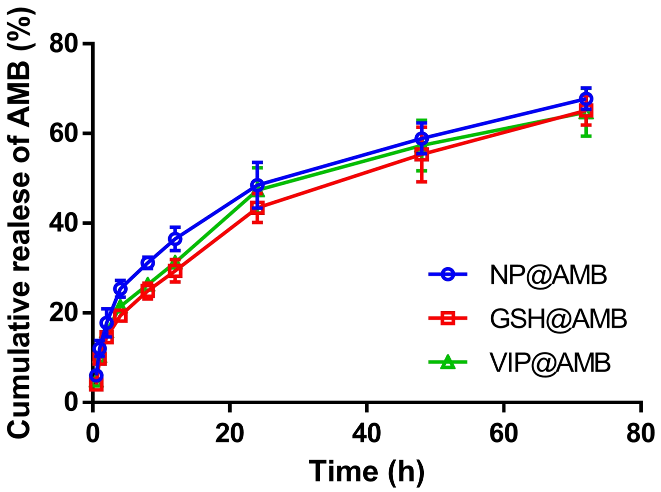

Supplementary Figure S18. *In vitro* cumulative release of amphotericin B (AMB) released from NP, GSH, or VIP@AMB. Data are presented as means ± SD (n=3 biologically independent samples). Source data are provided as a Source Data file.

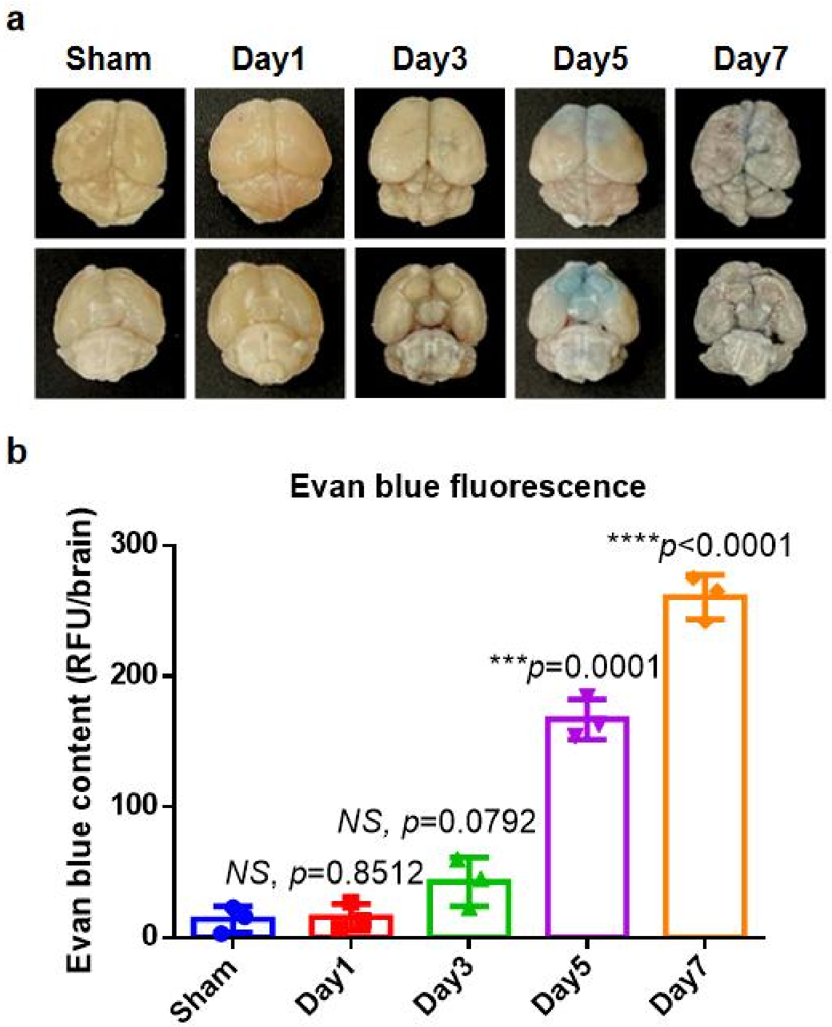

Supplementary Figure S19. Evans blue (EB) dye extravasation to determine blood-brain barrier integrity in female mice with cryptococcal *C neoformans* meningitis. (**a**) Images of Evans blue leakage from vessels in the brains of mice on day 1, 3, 5, 7 after infection. (**b**) Quantitative analysis of Evans blue leakage. Data are presented as means ± SD (n=3 biologically independent samples). ***$P<0.001$, ****$P<0.0001$, *NS* means no significance. Statistical significance was calculated with two-tailed unpaired *t* tests. Source data are provided as a Source Data file.

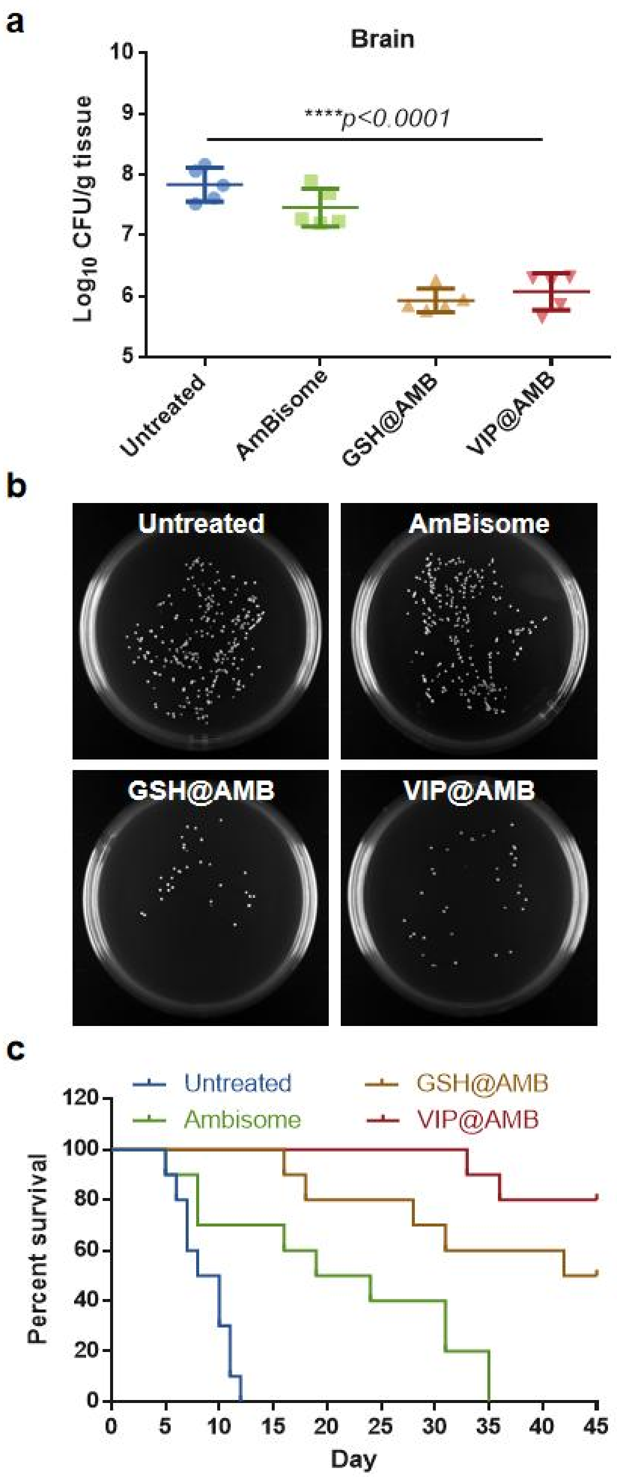

Supplementary Figure S20. (**a-b**) Fungal tissue burden in mice treated with the PBS, AmBisome, GSH@AMB, and VIP@AMB at day 6 after treatment in mice infected with *C. neoformans*-Luc. Data are presented as means ± SD (n=5 biologically independent samples). ****P<0.0001. Statistical significance was calculated with two-tailed unpaired t tests. Data are representative of two independent experiments with similar results. (**c**) Survival curves for each treatment group (n =10 biologically independent animals). Source data are provided as a Source Data file.

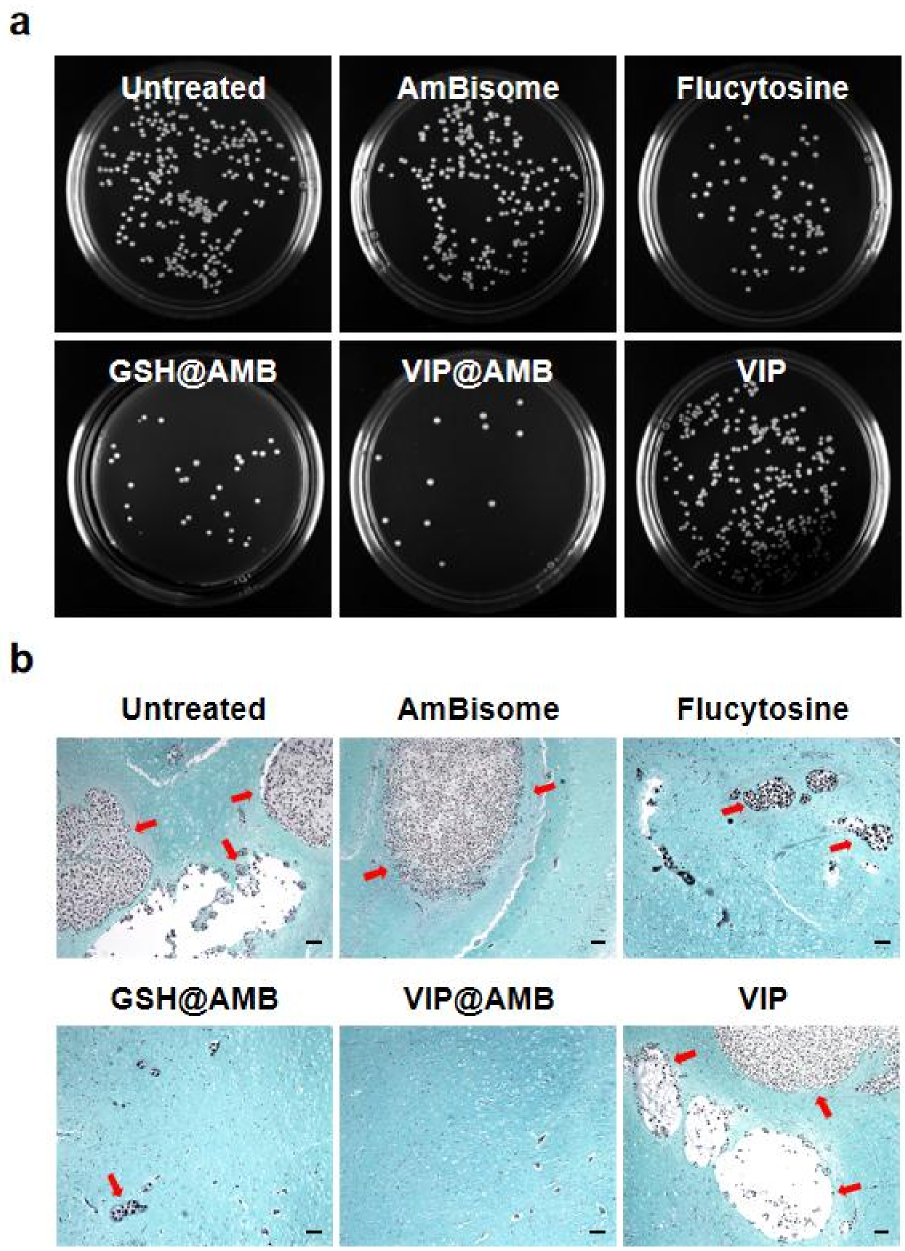

Supplementary Figure S21. (**a**) Brain tissue burden in mice treated with the PBS, AmBisome, flucytosine, GSH@AMB, VIP@AMB, or VIP at day 6 after treatment in mice infected with *C. neoformans*. (**b**) Brain sections after the treatment stained with Groctt-Gomori methenamine silver; *C. neoformans* appears as black. Scale bar = 50 µm. Data are representative of two independent experiments with similar results.

Supplementary Tables

Supplementary Table S1. Molar ratios of each component in the VIP formulations tested.

Name	Molar Ratio					
	Ionizable lipidoid	Helper lipid			Cholesterol	DMG-PEG$_{2000}$
		DOPE	DOPC	DSPC		
1	40	10	-	-	49	1
2	40	12.5	-	-	46	1.5
3	40	15	-	-	43	2
4	40	-	10	-	49	1
5	40	-	12.5	-	46	1.5
6	40	-	15	-	43	2
7	40	-	-	10	49	1
8	40	-	-	12.5	46	1.5
9	40	-	-	15	43	2
10	50	10	-	-	39	1
11	50	12.5	-	-	36	1.5
12	50	15	-	-	33	2
13	50	-	10	-	39	1
14	50	-	12.5	-	36	1.5
15	50	-	15	-	33	2
16	50	-	-	10	39	1
17	50	-	-	12.5	36	1.5
18	50	-	-	15	33	2
19	60	10	-	-	29	1
20	60	12.5	-	-	26	1.5
21	60	15	-	-	23	2
22	60	-	10	-	29	1
23	60	-	12.5	-	26	1.5
24	60	-	15	-	23	2
25	60	-	-	10	29	1
26	60	-	-	12.5	26	1.5
27	60	-	-	15	23	2

Supplementary Table S2. Physiochemical characterizations of VIP based on A1-B1-C2.1.

Name	Size, nm	Polydispersity Index
A1-B1-C2.1-1	170.6	0.275
A1-B1-C2.1-2	112.5	0.213
A1-B1-C2.1-3	168.6	0.302
A1-B1-C2.1-4	110.0	0.192
A1-B1-C2.1-5	313.9	0.442
A1-B1-C2.1-6	114.6	0.221
A1-B1-C2.1-7	>1000	—
A1-B1-C2.1-8	111.9	0.299
A1-B1-C2.1-9	>1000	—
A1-B1-C2.1-10	108.2	0.228
A1-B1-C2.1-11	171.6	0.260
A1-B1-C2.1-12	117.1	0.187
A1-B1-C2.1-13	175.6	0.320
A1-B1-C2.1-14	88.4	0.182
A1-B1-C2.1-15	132.0	0.322
A1-B1-C2.1-16	127.3	0.239
A1-B1-C2.1-17	117.9	0.276
A1-B1-C2.1-18	128.9	0.253
A1-B1-C2.1-19	139.8	0.284
A1-B1-C2.1-20	>1000	—
A1-B1-C2.1-21	136.7	0.26
A1-B1-C2.1-22	167.5	0.276
A1-B1-C2.1-23	115.7	0.226
A1-B1-C2.1-24	>1000	—
A1-B1-C2.1-25	128.7	0.285
A1-B1-C2.1-26	125.0	0.285
A1-B1-C2.1-27	187.4	0.292

Supplementary Table S3. Particle size and polydispersity index of VIP based on formulation 14.

Name	Size, nm	Polydispersity Index	Name	Size, nm	Polydispersity Index
A1-B1-C1.1	104.6	0.243	A3-B2-C1.1	207.0	0.285
A1-B1-C2.1	88.4	0.182	A3-B2-C2.1	171.4	0.389
A1-B1-C3.1	133.3	0.31	A3-B2-C3.1	134.3	0.325
A1-B1-C3.2	89.3	0.213	A3-B2-C3.2	87.7	0.224
A1-B1-C3.3	90.5	0.189	A3-B2-C3.3	122.7	0.337
A1-B1-C4.1	114.6	0.221	A3-B2-C4.1	>1000	—
A1-B1-C4.2	92.6	0.225	A3-B2-C5.1	145.6	0.298
A1-B1-C5.1	247.5	0.298	A4-B1-C1.1	157.0	0.357
A1-B2-C1.1	167.6	0.359	A4-B1-C2.1	139.0	0.294
A1-B2-C2.1	290.7	0.333	A4-B1-C3.1	143.9	0.249
A1-B2-C3.1	137.6	0.254	A4-B1-C3.2	160.0	0.393
A1-B2-C3.2	86.0	0.237	A4-B1-C3.3	167.7	0.305
A1-B2-C3.3	139.5	0.266	A4-B1-C4.1	141.8	0.361
A1-B2-C4.1	>1000	—	A4-B1-C4.2	115.9	0.263
A1-B2-C5.1	186.2	0.286	A4-B1-C5.1	141.8	0.361
A2-B1-C1.1	173.8	0.307	A4-B2-C1.1	>1000	—
A2-B1-C2.1	96.3	0.216	A4-B2-C2.1	144.6	0.331
A2-B1-C3.1	115.7	0.226	A4-B2-C3.1	156.9	0.419
A2-B1-C3.2	132.5	0.232	A4-B2-C3.2	94.0	0.227
A2-B1-C3.3	133.9	0.255	A4-B2-C3.3	107.4	0.184
A2-B1-C4.1	127.4	0.296	A4-B2-C4.1	103.6	0.269
A2-B1-C4.2	115.9	0.237	A4-B2-C5.1	91.3	0.228
A2-B1-C5.1	89.6	0.201	A5-B1-C1.1	94.9	0.482
A2-B2-C1.1	126.4	0.361	A5-B1-C2.1	124.1	0.358
A2-B2-C2.1	>1000	—	A5-B1-C3.1	128.3	0.315
A2-B2-C3.1	134.2	0.271	A5-B1-C3.2	86.2	0.270
A2-B2-C3.2	92.7	0.252	A5-B1-C3.3	91.0	0.235
A2-B2-C3.3	127.9	0.364	A5-B1-C4.1	134.1	0.370
A2-B2-C4.1	222.6	0.340	A5-B1-C4.2	88.7	0.113
A2-B2-C5.1	>1000	—	A5-B1-C5.1	104.7	0.170
A3-B1-C1.1	220.6	0.293	A5-B2-C1.1	100.4	0.240
A3-B1-C2.1	208.5	0.295	A5-B2-C2.1	>1000	—
A3-B1-C3.1	212.3	0.284	A5-B2-C3.1	114.7	0.206
A3-B1-C3.2	87.2	0.201	A5-B2-C3.2	121.0	0.224
A3-B1-C3.3	88.1	0.252	A5-B2-C3.3	105.4	0.252
A3-B1-C4.1	141.0	0.170	A5-B2-C4.1	113.5	0.175
A3-B1-C4.2	98.5	0.235	A5-B2-C5.1	175.0	0.227
A3-B1-C5.1	126.9	0.411			

Supplementary Table S4. Binding affinity (K_D) of three VIP compounds between PDE1 and bovine serum albumin (BSA) protein.

VIP Compound	PDE1	BSA
A1-B1-C3.3	7.02×10^{-7}	2.57×10^{-4}
A3-B1-C3.2	1.16×10^{-7}	1.65×10^{-5}
A5-B1-C4.2	6.29×10^{-8}	1.34×10^{-3}

Values shown are mol/L.

Supplementary Table S5. Molar ratios of each component in the indicated formulations.

	Helper Lipid	Cholesterol	DMG-PEG$_{2000}$	Ionizable Lipid	DSPE-PEG-GSH
NP	62.5	36	1.5	—	—
VIP	12.5	36	1.5	50	—
MC3	10	38.5	1.5	50	—
GSH	62.5	36	—	—	1.5

Abbreviations: NP, nanoparticle; VIP, vinpocetine-derived ionizable-lipidoid nanoparticle; MC3, commercially available lipid nanoparticle; GSH, GSH-LNP in which glutathione [GSH] was used as the target moiety.

Supplementary Table S6. Mechanisms, doses, and efficiency of the four agents.

Agent	Mechanism and Reference	Route of Administration	Dose	Ratio[a]
5-hydroxytryptamine (5-HT)	Vasoconstrictor[8]	lntravenous	500 μg/kg	↓ 36%
		Intrathecal	100 μg/kg	↓ 43%
Dexmedetomidine (Dex)	α2 receptor agonist[9]	lntravenous	10 μg/kg	↓ 42%
		Intrathecal	5 μg/kg	↓ 31%
N^G-monomethyl-L-arginine (L-NMMA)	NO synthase inhibitor[10]	lntravenous	7.5 mg/kg	↓ 36%
		Intrathecal	5 mg/kg	↓ 36%
Noradrenaline (NA)	α1 receptor agonist[11]	Intrathecal	1 mg/kg	↓ 35%

[a]Reduced ratio of cerebral blood flow for 60 min after a single dose of the agent.

Supplementary Table S7. Hematotoxicity analyses.

		PBS			VIP		
	**Range**	**1**	**2**	**3**	**4**	**5**	**6**
White blood cells, 10^9/L	2.61–13	3.86	2.83	4.04	4.83	5.04	6.37
Neutrophils, %	1.2–30	15.5	22.8	19.1	22.8	11.9	11.7
Lymphocytes, %	70–96	84.4	77.1	80.8	77.1	88	88.2
Monocytes, %	0–10	0	0	0	0	0	0
Eosinophils, %	0–10	0	0	0.1	0	0	0
Basophils, %	0–5	0.1	0.1	0	0.1	0.1	0.1
Neutrophils, 10^9/L	0.1–2	0.6	0.64	0.77	1.1	0.6	0.75
Lymphocytes, 10^9/L	1.27–8.44	3.26	2.19	3.27	3.73	4.44	5.62
Monocytes, 10^9/L	0–0.29	0	0	0	0	0	0
Eosinophils, 10^9/L	0–0.17	0	0	0	0	0	0
Basophils, 10^9/L	0–0.02	0	0	0	0	0	0
RBC, 10^9/L	6–12.5	6.93	7.13	6.7	7.66	7.87	8.63
Hb, g/L	100–190	121	121	117	132	128	159
Hematocrit, %	40–48	34.7	32.6	32.1	33.9	36	42.8
Mean corpuscular volume, fL	41–63	50	45.7	47.9	44.2	45.7	49.6
Mean corpuscular Hb, pg	13–19	17.5	16.9	17.5	17.2	16.3	18.4
Mean corpuscular Hb concentration, g/L	290–351	350	371	365	389	357	371
RBC distribution width CV, %	10–20	14.6	13.5	14.3	13.1	13.3	13.2
RBC distribution width SD, fL	0.1–99.9	30	25.3	28.2	23.6	24.5	26
Platelets, 10^9/L	540–1540	836	823	576	667	663	603
Mean platelet volume, fL	3.8–14.1	5.9	6.1	6.7	6.6	7.1	7
Platelet distribution width, %	0.1–30	6.2	5.7	7.3	7.2	9.2	8.3
Procalcitonin, %	0.01–9.99	0.497	0.505	0.386	0.44	0.469	0.42

Abbreviations: PBS, phosphate-buffered saline; VIP, vinpocetine-derived ionizable-lipidoid nanoparticle; the numbers (1-6), individual mice treated with PBS or VIP; RBC, red blood cells; Hb, hemoglobin, CV, coefficient of variation; SD, standard deviation.

Supplementary Table S8. Encapsulation efficiency of amphotericin B (AMB) in different preparations.

	**Encapsulation Efficiency (%)**
NP@AMB	82.72 ± 1.59
GSH@AMB	81.53 ± 3.50
VIP@AMB	84.12 ± 3.18

Abbreviations: NP, nanoparticle; GSH, GSH-LNP in which glutathione [GSH] was used as the target moiety; VIP, vinpocetine-derived ionizable-lipidoid nanoparticle.

Supplementary Table S9. *In vitro* minimum inhibitory concentration (MIC) of different formulations and compounds on *C. neoformans* and *C. neoformans*-Luc.

**MIC, μg/mL**	***C. neoformans***	***C. neoformans*-Luc**
AMB	0.5	0.5
AmBisome	0.5	0.5
Flucytosine	8	8
NP@AMB	0.5	0.5
GSH@AMB	0.5	0.5
VIP@AMB	0.5	0.5
VIP	>1024	>1024
A5-B1-C4.2	>1024	>1024

Abbreviations: AMB, amphotericin B; NP, nanoparticle; GSH, GSH-LNP in which glutathione [GSH] was used as the target moiety; VIP, vinpocetine-derived ionizable-lipidoid nanoparticle.

Supplementary references

1. Dunkern, T.R. & Hatzelmann, A. Characterization of inhibitors of phosphodiesterase 1C on a human cellular system. *The FEBS journal* **274**, 4812-4824 (2007).
2. Davidson, R.C. *et al.* Gene disruption by biolistic transformation in serotype D strains of Cryptococcus neoformans. *Fungal Genet Biol* **29**, 38-48 (2000).
3. Trott, O. & Olson, A.J. AutoDock Vina: improving the speed and accuracy of docking with a new scoring function, efficient optimization, and multithreading. *J Comput Chem* **31**, 455-461 (2010).
4. Delano, W.L. PyMOL: An Open-Source Molecular Graphics Tool. (2002).
5. Ravindranath, P.A., Forli, S., Goodsell, D.S., Olson, A.J. & Sanner, M.F. AutoDockFR: Advances in Protein-Ligand Docking with Explicitly Specified Binding Site Flexibility. *PLoS Comput Biol* **11**, e1004586 (2015).
6. Assmann, J.C. *et al.* Isolation and Cultivation of Primary Brain Endothelial Cells from Adult Mice. *Bio-protocol* **7** (2017).
7. Zhang, Y., Huo, M., Zhou, J. & Xie, S. PKSolver: An add-in program for pharmacokinetic and pharmacodynamic data analysis in Microsoft Excel. *Comput Methods Programs Biomed* **99**, 306-314 (2010).
8. Wang, B., Zhang, A.X., Zou, Y., Wang, J. & Xiao, J.G. [8-(N,N-diethylamino)-n-octyl-3,4,5-trimethoxybenzoate inhibited the reduction of cerebral blood flow evoked by 5-HT and KCl in rats]. *Yao Xue Xue Bao* **38**, 342-345 (2003).
9. Drummond, J.C. *et al.* Effect of dexmedetomidine on cerebral blood flow velocity, cerebral metabolic rate, and carbon dioxide response in normal humans. *Anesthesiology* **108**, 225-232 (2008).
10. White, R.P., Deane, C., Vallance, P. & Markus, H.S. Nitric oxide synthase inhibition in humans reduces cerebral blood flow but not the hyperemic response to hypercapnia. *Stroke* **29**, 467-472 (1998).
11. Froese, L., Dian, J., Gomez, A., Unger, B. & Zeiler, F.A. The cerebrovascular response to norepinephrine: A scoping systematic review of the animal and human literature. *Pharmacol Res Perspect* **8**, e00655 (2020).

12. Zhang, X. *et al.* Targeting pyroptosis with nanoparticles to alleviate neuroinflammatory for preventing secondary damage following traumatic brain injury. *Science advances* **10**, eadj4260 (2024).

13. Goldim, M.P.S., Della Giustina, A. & Petronilho, F. Using Evans Blue Dye to Determine Blood-Brain Barrier Integrity in Rodents. *Curr Protoc Immunol* **126**, e83 (2019).
