# Supplementary material for: Regulation of cerebral blood flow boosts precise brain targeting of vinpocetine-derived ionizable-lipidoid nanoparticles
Source: Nat Commun. 2024 May 11;15:3987. doi: 10.1038/s41467-024-48461-4 (PMC11088666; doi:10.1038/s41467-024-48461-4)
Supplement: Supplementary file 3 — Reporting Summary [file 41467_2024_48461_MOESM3_ESM.pdf]

nature portfolio

Corresponding author(s): Wen Jiang, Zhaogang Yang, Chong Li

Last updated by author(s): 19 th April, 2024

Reporting Summary

Nature Portfolio wishes to improve the reproducibility of the work that we publish. This form provides structure for consistency and transparency in reporting. For further information on Nature Portfolio policies, see our Editorial Policies and the Editorial Policy Checklist.

Please do not complete any field with "not applicable" or n/a. Refer to the help text for what text to use if an item is not relevant to your study.
For final submission: please carefully check your responses for accuracy; you will not be able to make changes later.

Statistics

For all statistical analyses, confirm that the following items are present in the figure legend, table legend, main text, or Methods section.

n/a	Confirmed	
☐	☒	The exact sample size (n) for each experimental group/condition, given as a discrete number and unit of measurement
☐	☒	A statement on whether measurements were taken from distinct samples or whether the same sample was measured repeatedly
☐	☒	The statistical test(s) used AND whether they are one- or two-sided<br>*Only common tests should be described solely by name; describe more complex techniques in the Methods section.*
☒	☐	A description of all covariates tested
☒	☐	A description of any assumptions or corrections, such as tests of normality and adjustment for multiple comparisons
☐	☒	A full description of the statistical parameters including central tendency (e.g. means) or other basic estimates (e.g. regression coefficient) AND variation (e.g. standard deviation) or associated estimates of uncertainty (e.g. confidence intervals)
☐	☒	For null hypothesis testing, the test statistic (e.g. F, t, r) with confidence intervals, effect sizes, degrees of freedom and P value noted<br>*Give P values as exact values whenever suitable.*
☒	☐	For Bayesian analysis, information on the choice of priors and Markov chain Monte Carlo settings
☒	☐	For hierarchical and complex designs, identification of the appropriate level for tests and full reporting of outcomes
☒	☐	Estimates of effect sizes (e.g. Cohen's d, Pearson's r), indicating how they were calculated

Our web collection on statistics for biologists contains articles on many of the points above.

Software and code

Policy information about availability of computer code

Data collection	FACSverse; Rayme multimode analyser; Operetta CLS; Lumina III Imaging; Smart-LF; EthO Vision XT8.5; Archimed; DMi8
Data analysis	GraphPad Prism version 8; FlowJo version 7.6; Tracedrawer evaluation software; Tanon gel image analysis system ; Imaris; ImageJ; Zeiss ZEN; AutoDock Vina 1.1.2; PyMol 2.5.4; ADFRsuite 1.0; PKsolver 2.0.10

For manuscripts utilizing custom algorithms or software that are central to the research but not yet described in published literature, software must be made available to editors and reviewers. We strongly encourage code deposition in a community repository (e.g. GitHub). See the Nature Portfolio guidelines for submitting code & software for further information.

Data

Policy information about availability of data

All manuscripts must include a data availability statement. This statement should provide the following information, where applicable:
- Accession codes, unique identifiers, or web links for publicly available datasets
- A description of any restrictions on data availability
- For clinical datasets or third party data, please ensure that the statement adheres to our policy

The data supporting the findings of this study are available within the paper and its Supplementary Information. Detailed information are provided in the Supplementary Materials and Methods, Supplementary Figs. 1 – 21, Supplementary Tables 1 – 9, and the Source Data file. Source data are provided with this paper

Research involving human participants, their data, or biological material

Policy information about studies with human participants or human data. See also policy information about sex, gender (identity/presentation), and sexual orientation and race, ethnicity and racism.

Reporting on sex and gender	No human research was invovled
Reporting on race, ethnicity, or other socially relevant groupings	
Population characteristics	
Recruitment	
Ethics oversight	

Note that full information on the approval of the study protocol must also be provided in the manuscript.

Field-specific reporting

Please select the one below that is the best fit for your research. If you are not sure, read the appropriate sections before making your selection.

☒ Life sciences ☐ Behavioural & social sciences ☐ Ecological, evolutionary & environmental sciences

For a reference copy of the document with all sections, see nature.com/documents/nr-reporting-summary-flat.pdf

Life sciences study design

All studies must disclose on these points even when the disclosure is negative.

Sample size	The chosen sample size was determined to ensure sufficient statistical significance while adhering to principles of animal welfare, with a particular emphasis on the 3R approach (replacement, reduction, and refinement).
Data exclusions	No animals were excluded from the final analysis.
Replication	The number of repetitions for the experiments has been described in the figure legends. Data are shown as mean with standard deviation.
Randomization	In this study, samples were allocated into experimental groups using a randomization procedure.
Blinding	The investigators were not blinded to the randomization, but were blinded to the data collection and final analysis.

Behavioural & social sciences study design

All studies must disclose on these points even when the disclosure is negative.

Study description	
Research sample	
Sampling strategy	
Data collection	
Timing	
Data exclusions	
Non-participation	
Randomization	

Ecological, evolutionary & environmental sciences study design

All studies must disclose on these points even when the disclosure is negative.

Study description	
Research sample	
Sampling strategy	
Data collection	
Timing and spatial scale	
Data exclusions	
Reproducibility	
Randomization	
Blinding	

Did the study involve field work? ☐ Yes ☐ No

Field work, collection and transport

Field conditions	
Location	
Access & import/export	
Disturbance	

Reporting for specific materials, systems and methods

We require information from authors about some types of materials, experimental systems and methods used in many studies. Here, indicate whether each material, system or method listed is relevant to your study. If you are not sure if a list item applies to your research, read the appropriate section before selecting a response.

Materials & experimental systems

n/a	Involved in the study	
☐	☒	Antibodies
☐	☒	Eukaryotic cell lines
☒	☐	Palaeontology and archaeology
☐	☒	Animals and other organisms
☒	☐	Clinical data
☒	☐	Dual use research of concern
☒	☐	Plants

Methods

n/a	Involved in the study	
☒	☐	ChIP-seq
☐	☒	Flow cytometry
☒	☐	MRI-based neuroimaging

Antibodies

Antibodies used

PDE1 monoclonal Antibody, Cat.# sc-376474 was purchased from Santa Cruz Biotechnology. BACE1 monoclonal antibody, Cat.# ab183612 was purchased from Abcam. Phospho-Tau (Ser396) Polyclonal Antibody, Cat.# 44-752G and Phospho-GSK3β (Tyr216) Polyclonal Antibody, Cat.# 44-604G were purchased from Invitrogen. beta amyloid 1-16 Polyclonal antibody, Cat.# bs-10558R was purchased from Bioss Biotechnology Co., Ltd. GAPDH Polyclonal Antibody, Cat.# 10494-1-AP; Beta Actin Polyclonal antibody, Cat.# 20536-1-AP; Fluorescein (FITC) – conjugated Affinipure Goat Anti-Rabbit IgG(H+L), Cat.# SA00003-2 and HRP-conjugated Affinipure Goat Anti-Rabbit IgG (H+L), Cat.# SA00001-2 were purchased from Proteintech Group, Inc.

Validation

PDE1 monoclonal Antibody(sc-376474) : WB, IP, IF, IHC(P) , ELISA; BACE1 monoclonal antibody(ab183612) : IHC-P, WB, IP, IHC-Fr; Phospho-Tau (Ser396) Polyclonal Antibody(44-752G):IHC, WB, ICC/IF, ELISA; Phospho-GSK3β (Tyr216) Polyclonal Antibody(44-604G): WB; beta amyloid 1-16 Polyclonal antibody(bs-10558R):IHC-P, IHC-F, ICC, IF=, Flow-Cyt, ELISA; GAPDH Polyclonal Antibody(10494-1-AP): FC, IF/ICC, IHC, IP, WB, ELISA; Beta Actin Polyclonal antibody(20536-1-AP): FC, IF/ICC, IHC, WB, ELISA; Fluorescein (FITC) – conjugated Affinipure Goat Anti-Rabbit IgG(H+L)(SA00003-2) :IF, FC; HRP-conjugated Affinipure Goat Anti-Rabbit IgG (H+L)(SA00001-2): ELISA, WB, Dot blot.

Eukaryotic cell lines

Policy information about cell lines and Sex and Gender in Research

Cell line source(s)	bEnd.3 cells, A172 cells, and GL261-Luc cells were purchased from KeyGen Biotech (Jiangsu, China). Primary BMECs were obtained from SD rats (4-6 weeks old).
Authentication	The cell lines were authenticated by the supplier by using mycoplasma analysis, sterility analysis, species diserimination using CO1 barcode assay, and visual analysis for growth determination, morphology and viability.
Mycoplasma contamination	The cell lines for the in vitro experiments were not tested for mycoplasma contamination. The cells for in Vivo experiments were tested for mycoplasma contamination before transplantation, and they are nagtive.
Commonly misidentified lines (See ICLAC register)	No commonly misidentified cell lines were used.

Palaeontology and Archaeology

Specimen provenance	
Specimen deposition	
Dating methods	

☐ Tick this box to confirm that the raw and calibrated dates are available in the paper or in Supplementary Information.

Ethics oversight	

Note that full information on the approval of the study protocol must also be provided in the manuscript.

Animals and other research organisms

Policy information about studies involving animals; ARRIVE guidelines recommended for reporting animal research, and Sex and Gender in Research

Laboratory animals	Balb/c mice (5-6 weeks), C57BL/6 mice (8 months), Sprague Dawley rats (8 weeks) and APP-PS-1 mice (2,4, 6, 8months) were used.
Wild animals	The study did not involve wild animals.
Reporting on sex	In consideration of achieving gender balance across various animal models to ensure the universality of targeting and efficacy, we adopted male APP/PS1 mice for the AD model and female mice for the brain tumor and meningitis models to comprehensively evaluate the VIP system.
Field-collected samples	The study did not involve samples collected from the field.
Ethics oversight	The laboratory animal facility has been accredited by the IACUC (Institutional Animal Care and Use Committee) of Southwest University Laboratory Animal Center (IACUC Issue No. IACUC-20221114-14). All animal experiments were conducted under the guidelines of the Ethical Review Committee of Experimental Animals at the Southwest University of China.

Note that full information on the approval of the study protocol must also be provided in the manuscript.

Clinical data

Policy information about clinical studies

All manuscripts should comply with the ICMJE guidelines for publication of clinical research and a completed CONSORT checklist must be included with all submissions.

Clinical trial registration	
Study protocol	
Data collection	
Outcomes	

Dual use research of concern

Policy information about dual use research of concern

Hazards

Could the accidental, deliberate or reckless misuse of agents or technologies generated in the work, or the application of information presented in the manuscript, pose a threat to:

No | Yes
- [ ] [ ] Public health
- [ ] [ ] National security
- [ ] [ ] Crops and/or livestock
- [ ] [ ] Ecosystems
- [ ] [ ] Any other significant area

Experiments of concern

Does the work involve any of these experiments of concern:

No | Yes
- [ ] [ ] Demonstrate how to render a vaccine ineffective
- [ ] [ ] Confer resistance to therapeutically useful antibiotics or antiviral agents
- [ ] [ ] Enhance the virulence of a pathogen or render a nonpathogen virulent
- [ ] [ ] Increase transmissibility of a pathogen
- [ ] [ ] Alter the host range of a pathogen
- [ ] [ ] Enable evasion of diagnostic/detection modalities
- [ ] [ ] Enable the weaponization of a biological agent or toxin
- [ ] [ ] Any other potentially harmful combination of experiments and agents

Plants

Seed stocks

Novel plant genotypes

Authentication

ChIP-seq

Data deposition

- [ ] Confirm that both raw and final processed data have been deposited in a public database such as GEO.
- [ ] Confirm that you have deposited or provided access to graph files (e.g. BED files) for the called peaks.

Data access links
May remain private before publication.

Files in database submission

Genome browser session
(e.g. UCSC)

Methodology

Replicates

Sequencing depth

Antibodies

Peak calling parameters

Data quality

Software

Flow Cytometry

Plots

Confirm that:

- [ ] The axis labels state the marker and fluorochrome used (e.g. CD4-FITC).
- [x] The axis scales are clearly visible. Include numbers along axes only for bottom left plot of group (a 'group' is an analysis of identical markers).
- [ ] All plots are contour plots with outliers or pseudocolor plots.
- [ ] A numerical value for number of cells or percentage (with statistics) is provided.

Methodology

Sample preparation: After the required treatments and incubation, the cells were washed with PBS before collection. Unstained cells and isotype controls were used. Flow eytometry was performed on a BD FACSVerse flow cytometer (BD Biosciences, USA), and the data were analysed by using FlowJo (FLowJo LLC).

Instrument: BD FACSVerse

Software: Data were collected by using BD FACSVerse software and analysed by using FLowJo Version 7.6.

Cell population abundance: Cell sorting was not performed.

Gating strategy: Cells were initially on a dot plot, SSC-A vs. FSC-A. The negative population was determined by using unstained cells and isotype controls. All the cells outside the negative population were considered as positive cell population.

- [ ] Tick this box to confirm that a figure exemplifying the gating strategy is provided in the Supplementary Information.

Magnetic resonance imaging

Experimental design

Design type:

Design specifications:

Behavioral performance measures:

Imaging type(s):

Field strength:

Sequence & imaging parameters:

Area of acquisition:

Diffusion MRI: [ ] Used [ ] Not used

Preprocessing

Preprocessing software:

Normalization:

Normalization template:

Noise and artifact removal:

Volume censoring:

Statistical modeling & inference

Model type and settings:

Effect(s) tested:

Specify type of analysis: [ ] Whole brain [ ] ROI-based [ ] Both

Statistic type for inference

(See Eklund et al. 2016)

Correction

Models & analysis

n/a	Involved in the study
☐	☐ Functional and/or effective connectivity
☐	☐ Graph analysis
☐	☐ Multivariate modeling or predictive analysis

Functional and/or effective connectivity

Graph analysis

Multivariate modeling and predictive analysis

This checklist template is licensed under a Creative Commons Attribution 4.0 International License, which permits use, sharing, adaptation, distribution and reproduction in any medium or format, as long as you give appropriate credit to the original author(s) and the source, provide a link to the Creative Commons license, and indicate if changes were made. The images or other third party material in this article are included in the article's Creative Commons license, unless indicated otherwise in a credit line to the material. If material is not included in the article's Creative Commons license and your intended use is not permitted by statutory regulation or exceeds the permitted use, you will need to obtain permission directly from the copyright holder. To view a copy of this license, visit http://creativecommons.org/licenses/by/4.0/
